# Trehalose’s untapped mechanisms in alzheimer’s: gut–brain–autophagy signalling beyond the usual targets

**DOI:** 10.1007/s40199-026-00647-5

**Published:** 2026-08-31

**Authors:** Sathish Kumar Gunasekaran, Harshith Kariappa C. K., Mirunalini Gobinath, Parikshit Roychowdhury, Manjula S. N.

**Affiliations:** 1 JSS College of Pharmacy, JSSAHER, Mysuru, India; 2 JSS College of Pharmacy, JSSAHER, Ooty, India

**Keywords:** Trehalose, Alzheimer’s disease, Neuroprotection, Autophagy, Gut–brain axis, Microbiome modulation

## Abstract

**Background:**

Trehalose is a promising therapeutic candidate for Alzheimer’s disease (AD) that is known to induce autophagy and facilitate misfolded proteins clearance such as amyloid-β and hyperphosphorylated tau. Despite there is emerging evidence that trehalose has a wider range of molecular mechanisms and thus has a greater neuroprotective profile.

**Objective:**

To summarize the emerging molecular mechanisms underlying the neuroprotective effects of trehalose beyond classical autophagy and discuss its therapeutic potential in AD.

**Methods:**

Published evidence from preclinical studies including in-vitro and in-vivo models, along with hypothetical and emerging findings from early clinical investigations was reviewed to evaluate the molecular mechanisms, therapeutic effects, and translational challenges associated with trehalose in AD.

**Results:**

In addition to classical autophagy signalling, recent studies have demonstrated that autophagy can also regulate the stability of neuronal membrane microdomains, prevent lipid bilayers disruption by amyloid proteins, and regulate stress granules dynamics that affect the function of RNA-binding proteins. Other discoveries indicate that interactions with nutrient-sensing pathways and glucose transporter systems that simulate metabolic stress, which may activate protective mechanisms separate from the inhibition of mTOR. Trehalose could also involve in lysosomal-autophagosome fusion and modulate the microglia and astrocytes activation, suggesting an important immunometabolic function. Trehalose often connects to the gut-brain axis and show their effect in gut microbiota composition, microbial metabolite signalling and gut barrier function. These effects can influence systemic inflammation, availability of short-chain fatty acids, bile acid profiles and vagus-mediated gut-to-brain communication, all of which can influence neuroinflammation networks in AD.

**Conclusion:**

Although promising results have been reported, primarily from preclinical studies, with early human investigations now beginning to emerge, opportunities remain to address, such as poor oral bioavailability, penetration into the brain and long-term safety in elderly patients. Mechanistic dissection in multi-omics approaches, microbiome-stratified models and early-phase clinical testing are the areas that need to be targeted in future research. A broader understanding of the mechanisms of action of trehalose provides a good chance to reimagine its therapeutic implications and develop novel approaches to AD.

## Introduction

Alzheimer’s disease (AD) is the most common form of dementia worldwide and a significant contributor to disability and dependency in older people. The concept of AD as a purely amyloid-centric disease has evolved to a multifactorial neurodegenerative syndrome involving progressive deposition of amyloid-β (Aβ) and pathological tau, early synaptic dysfunction, network disintegration and neuroinflammation. Large integrative reviews emphasise that Aβ, hyperphosphorylated tau, calcium dysregulation and chronic inflammatory cascades interact in self-amplifying feed-forward loops that ultimately drive neuronal loss and cognitive decline [1, 2]. Soluble Aβ oligomers and misfolded tau species are now recognised as key mediators of synaptic failure, impairing glutamatergic signalling, plasticity and spine stability well before overt neuronal death [3, 4].

Despite the arrival of amyloid-targeting antibodies, clinical benefit remains modest and accompanied by safety concerns, underlining the need for complementary strategies that target upstream modulators of proteostasis, i.e., protein homeostasis network in cellular stress responses and systemic contributors such as metabolism and the gut-brain axis [5]. Defective protein quality control and autophagy-lysosome dysfunction are central to this broader view of AD pathogenesis. Autophagy genes and markers of lysosomal function are altered in post-mortem AD brain, and dystrophic neurites are enriched in autophagic vacuoles and undigested cargo, consistent with modulated autophagic flux [6].

**Fig. 1:**
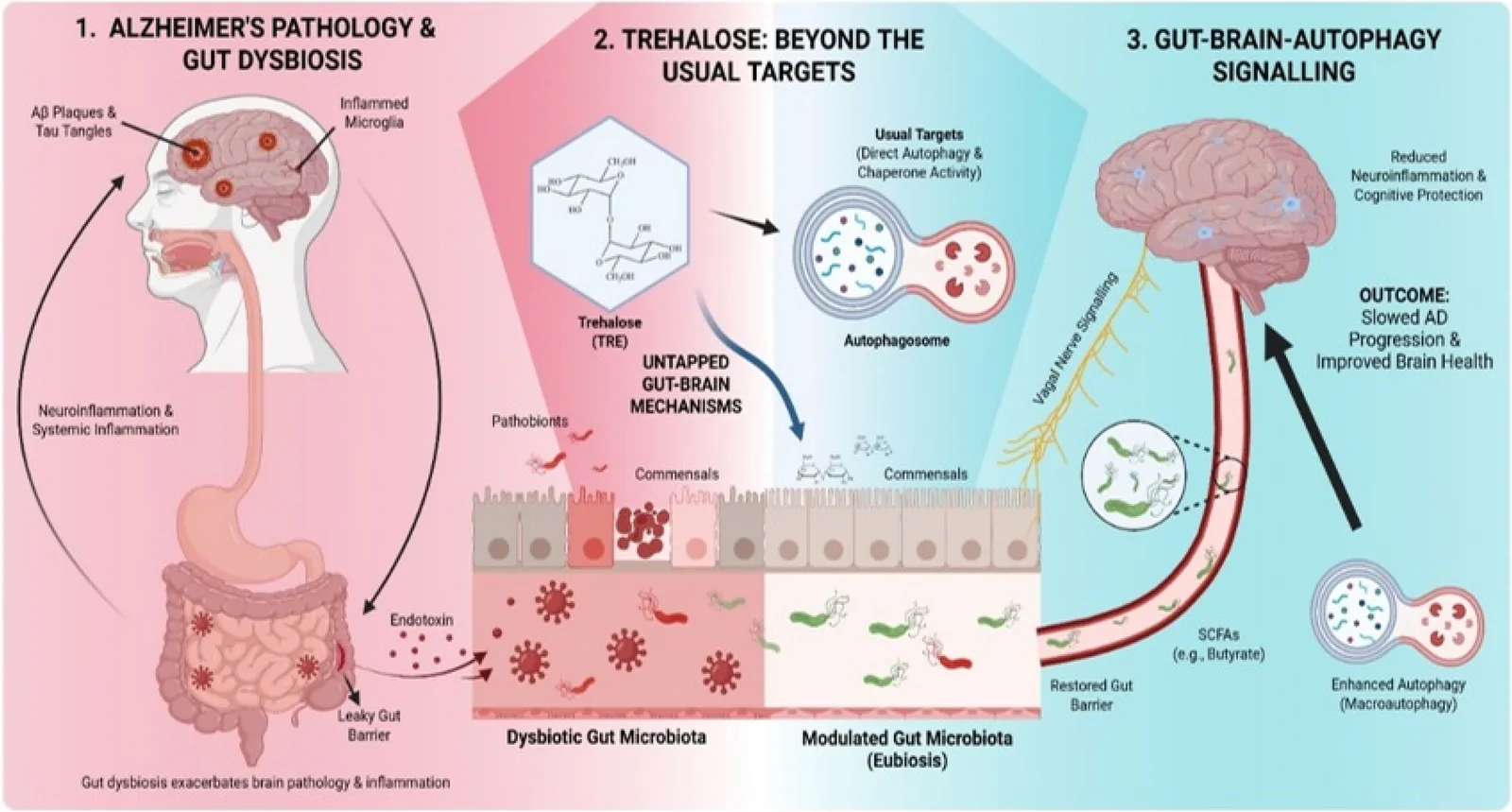
Proposed gut–brain–autophagy pathways linking trehalose to alzheimer’s disease

Experimental work shows that defective clearance of misfolded Aβ and tau through the autophagy-lysosome system accelerates aggregation and synaptic toxicity, whereas enhancing autophagy can attenuate pathology in multiple disease models [6, 7]. These observations have led to rising interest in small molecules that restore proteostasis, including the naturally occurring disaccharide trehalose. Trehalose (α-D-glucopyranosyl-α-D-glucopyranoside) is a non-reducing sugar widespread in bacteria, fungi, plants and invertebrates, where it functions as a stress-protective osmolyte and protein stabiliser. It is widely used in food and pharmaceutical formulations because of its ability to preserve protein structure and membrane integrity under desiccation, heat and oxidative stress [8, 9]. Over the past two decades, trehalose has attracted attention in neurology after the discovery that it can induce autophagy in an apparently mTOR-independent manner, promote the clearance of aggregation-prone proteins and ameliorate behavioural deficits in models of Huntington’s, Parkinson’s and AD [8, 10, 11]. Trehalose also ameliorates proteostasis, modulates inflammatory responses and reduces pathological protein burden across several neurodegenerative models [10]. In amyloid precursor protein (APP) and tau-based models, trehalose administration reduces Aβ and phospho-tau accumulation, improves synaptic markers and rescues deficits in and memory performance [12, 13]. At the cellular level, trehalose decreases secreted Aβ levels and alters APP processing in neuronal cultures, suggesting actions both on aggregate clearance and on upstream production pathways [13, 14]. Recent work in HT22 hippocampal cells exposed to Aβ_1−42_ oligomers showed that trehalose, unlike several other disaccharides, preserved cell viability, implicating specific neuroprotective properties beyond simple osmotic or caloric effects [15]. All these data have put trehalose as a promising target for proteostasis malfunction in AD. The mechanism of trehalose action is, however, controversial and likely to be more complex. Lee et al. suggested that the use of trehalose is not just a “turn on” for autophagy but can inhibit the autophagy pathway at its late stages in certain situations, which means the beneficial effects of trehalose might not be due to direct autophagic flux stimulation [14]. Similarly, Pupyshev et al. highlighted that trehalose has several effects that are relevant for neurodegeneration, such as modulation of oxidative stress, mitochondrial function and inflammatory signalling, and that its low penetration into the blood-brain barrier makes it difficult to interpret its effects solely on the neuronal level [6]. Such observations have led to a new perspective on trehalose as a pleiotropic regulator of cellular and systemic homeostasis [16]. New biophysical and cell-biological studies offer a first look at poorly studied molecular mechanisms by which trehalose can affect AD-relevant processes. At the membrane level, high-resolution biophysical measurements indicate that trehalose reduces the binding of Aβ to lipid bilayers, stabilises membrane structure and mitigates amyloid-induced disruption of model neuronal membranes [17, 18]. As an osmolyte and “chemical chaperone,” trehalose can modulate protein folding landscapes and retard fibrillation of model proteins, supporting the idea that direct effects on protein-water-lipid interactions contribute to its neuroprotective profile [9, 16]. Gut dysbiosis is linked to neuroinflammation, altered Aβ metabolism, microglial priming and BBB disruption, in part through microbially derived metabolites such as short-chain fatty acids and bile acids, as well as peripheral immune activation and vagus-mediated signalling [19, 20]. These insights have sparked interest in microbiome-targeted interventions including dietary components, prebiotics, probiotics and faecal microbiota transplantation as potential disease-modifying strategies in AD [19–21]. Trehalose sits at the intersection of these themes because it is ingested orally in most experimental and potential clinical applications, is extensively metabolised in the gut and can influence microbial communities. Early mechanistic work already speculated that trehalose might exert neuroprotective effects indirectly by shaping the gut microbiota [22, 23]. Subsequent studies in metabolic and neurodegenerative models demonstrated that trehalose intake alters gut microbiota composition, hepatic and systemic metabolic profiles and markers of gut-brain communication [16, 24]. Trehalose was found to have a systems-level mode of action in a Parkinson’s disease model with prominent gastrointestinal dysfunction by modulating the microbiota-gut-brain axis and preserving dopaminergic neurons in the gut and brain [25]. Complementary in vitro work shows that trehalose-derived galacto oligosaccharides selectively enrich beneficial genera such as Bifidobacterium and Lactobacillus while increasing production of butyrate, a short-chain fatty acid with recognised neuroactive and anti-inflammatory properties [26]. Taken together, these findings strengthen the hypothesis that part of trehalose’s neuroprotective potential may be mediated through modulation of the microbiota-gut-brain axis rather than through direct action on central neurons alone. Despite a growing number of reviews on trehalose and neurodegeneration, most accounts still frame its role primarily in terms of autophagy induction and aggregate clearance [12, 23]. Much less attention has been paid to the emerging, and sometimes contradictory, data on trehalose’s effects on membrane biophysics, nutrient-sensing and transporter pathways, lysosomal competence, glial immunometabolism and gut-mediated signalling relevant to AD. Likewise, the implications of limited brain penetration, context-dependent autophagy modulation and individual variability in gut microbiota composition for the clinical translation of trehalose remain underexplored [8, 24, 27]. This review aims to provide an updated and integrative overview of trehalose in the context of AD, with a particular focus on these underrated mechanistic insights.

 (Fig. 1) The schematic illustrates established and putative mechanisms through which trehalose may influence Alzheimer’s disease (AD) pathology along the gut–brain–autophagy axis. On the left, gut dysbiosis and impaired intestinal barrier function can promote systemic inflammation and neuroinflammation, thereby contributing to amyloid-β and tau-associated AD pathology. In the center, trehalose is depicted as acting through its established autophagy-modulating effects together with emerging, comparatively underexplored actions on the gut microbiota and intestinal environment. On the right, trehalose-associated modulation of gut homeostasis may influence gut–brain communication through microbial metabolites, immune signalling, and vagal pathways, while its autophagy-related actions may facilitate neuronal proteostasis and attenuation of neuroinflammatory processes. Collectively, these interconnected mechanisms are hypothesized to contribute to neuroprotection and potentially modify AD-related pathological progression.

## Trehalose: structure, biochemistry and pharmacokinetics

### Chemical and physicochemical properties

Trehalose is a non-reducing disaccharide composed of two α-D-glucose units linked by an α, α−1,1-glucosidic bond [22, 28–30] because both anomeric carbons of the glucose residues are involved in the linkage, it lacks a free reducing end, which confers high chemical stability (resistance to acid hydrolysis and enzymatic cleavage) compared with many other sugars [29, 30]. From a physicochemical perspective, trehalose’s unique bond gives it several distinct properties including high hydrophilicity, capacity to form hydrogen‐bond networks with water, substantial osmoprotective capacity in desiccation or stress conditions, and an ability to stabilise proteins and lipid membranes [28, 31]. For example, molecular dynamics and Raman investigations have revealed that trehalose solutions retard the dynamics of water molecules and create structured hydration shells, which explain its ability to protect proteins from denaturation [30, 32]. These properties make trehalose a “chemical chaperone” in various biological and biotechnological applications [30, 31]. The anhydrous molecular weight is ~ 342 g/mol (C_₁₂_H_₂₂_O_₁₁_) and the solubility in water is high but varies with temperature and hydration. The non‐reduction is also associated with a lower number of interactions through glycation pathways than reducing sugars [29, 30]. To conclude, the chemical structure of trehalose is responsible for a series of biophysical & biochemical characteristics such as chemical inertness, hydration shell formation, osmotic protection, membrane & protein stabilisation that are desirable in cellular stress and therapeutic studies.

### Absorption, metabolism, and systemic distribution

Trehalose is digested in the small intestine by the brush-border enzyme trehalase, which breaks it down to two molecules of glucose when taken orally. Mammals cannot synthesise trehalose, but the enzyme trehalase is expressed in the intestinal epithelium [27, 28]. In a human digestion and absorption study, trehalose was absorbed and digested at a slower rate than some other sugars, probably because of the additional enzymatic step [33, 34]. Very little information has been reported about the complete pharmacokinetics of trehalose in humans, such as its absorption, peak plasma concentrations, and half‐life. Preliminary animal studies indicate that following ingestion, some trehalose may bypass metabolism and reach systemic circulation (i.e., not be hydrolysed immediately), while there is limited penetration to the brain [23, 27]. Little is known about trehalose distribution outside the gut, although some research suggests it can be absorbed by peripheral tissues, but with low or variable BBB permeability [35]. The resulting glucose is then used for normal carbohydrate metabolism. In mammals, there is little evidence of biochemical transformation of trehalose per se other than hydrolysis in the gut. Unlike in organisms that synthesise trehalose for stress protection, in mammals the disaccharide is effectively a cosmetic or nutritional sugar unless specific delivery or protection strategies are used [27, 28]. From a neurotherapeutic angle, the limited systemic and brain exposure raises questions about how trehalose exerts protective effects in central nervous system (CNS) models whether via direct neuronal uptake or indirectly via peripheral/gut‐mediated mechanisms [27, 36].

### Stability, dosing considerations and delivery challenges

Trehalose is chemically stable in solution, under heat, acid and oxidative stress conditions compared to many sugars, which is one reason it’s used in food, pharmaceutical and biotech applications [29, 30]. In biological settings, its use as a therapeutic agent raises several key considerations. Preclinical models of neurodegenerative disease often use high concentrations (for example, in drinking water or diet) or parenteral administration. For example, animal studies in AD models have used 2% to 4% trehalose solutions in drinking water to obtain neuroprotective effects [37]. This is difficult to convert into human equivalent dosage for both safety and practicality. Trehalose may undergo substantial pre-systemic hydrolysis by intestinal trehalase, with a consequent decrease in the availability of intact trehalose. Additionally, delivery to the brain is limited by BBB and low passive permeability. Interest is growing in the use of improved delivery systems, such as carriers containing trehalose or polymer conjugates or formulation techniques to avoid metabolic degradation [38]. Chemical stability is reasonably guaranteed, but biological stability (enzymatic hydrolysis and metabolic clearance) is not as certain. Metabolic effects (e.g., glucose load) may confound evaluation of neuroprotective effects with repeated or high doses. In the past few years, however, safety studies in rodents have demonstrated that neuroprotective doses of trehalose are tolerated and do not significantly alter the architecture of metabolic organs [34]. One of the main translational barriers is the difference between the doses needed to see CNS effects in animals and the practical dose limitation in humans. Additionally, it is still challenging to differentiate central (neuronal uptake) and peripheral (gut microbiome modulation, systemic metabolism) effects of trehalose. The chemical stability of trehalose is good, and its neuroprotective properties are interesting, but the pharmacokinetic and delivery challenges are not insignificant. Proper formulation, dosing regimen and mechanism‐of‐action research are essential to further the translational application.

## Established neuroprotective mechanisms of trehalose

### Autophagy induction and proteostasis regulation

One of the principal mechanisms attributed to trehalose is modulation of autophagy, the cellular process that is responsible for the degradation and recycling of damaged proteins and organelles, thereby improving proteostasis in neuronal cells. Early studies have reported elevated LC3 II/LC3 I ratios, reduced levels of p62/SQSTM1, and increased autophagosome formation following to trehalose treatment in models of neurodegenerative disease [13, 23, 39]. In experimental models of AD, trehalose treatment was also associated with reduced amyloid-β (Aβ) and tau aggregation, which is consistent with an autophagy-mediated proteostatic clearance mechanism [23]. In contrast, some studies suggest that though autophagy-like markers increase, late-stage flux of autophagosome to autolysosome fusion and cargo degradation may be impaired under certain experimental conditions [13, 39]. These controversies indicate that the effects of trehalose on autophagy may depend on factors such as cell type, metabolic state, lysosomal function, and experimental models. In neuron-based models, trehalose has an additional activity of upregulating chaperone proteins, enhance lysosomal enzyme activity, and stabilize misfolded protein conformations, thereby reducing the stress on proteostasis networks [13]. Therefore, these findings suggest that trehalose appears to engage in multiple components of the proteostasis mechanisms, although its precise effects on autophagic flux likely vary according to cellular and disease context [40].

### Clearance of amyloid-β, tau and misfolded proteins

The salient characteristics in the context of AD pathology are extracellular Aβ plaques, intracellular tau tangles and widespread synaptic dysfunction. Trehalose has been found to have a consistent effect on reducing Aβ levels, decreasing phosphorylated-tau species and maintaining markers of synapses in animal models. Transgenic APP/PS1 mice fed trehalose showed marked decreases in plaque load and enhanced memory performance in memory tasks. The clearance could occur through an increase in autophagic clearance of aggregated proteins, and/or a decrease in the production of misfolded proteins (possibly through chaperone stabilization or by inhibition of aggregation nucleation). Other research indicates that trehalose also can regulate APP processing, which decreases the production of Aβ [13, 23]. The results support the hypothesis that trehalose could have a modulatory effect on the disease processes that are not only downstream effects of the disease but also processes that drive the disease.

### Anti-oxidative and anti-inflammatory effects

In addition to proteostasis, trehalose has neuroprotective effects through oxidative stress and inflammatory pathways. In models of dopaminergic neurons, trehalose promoted nuclear translocation of Nrf2, a master regulator of antioxidant defenses, increased glutathione peroxidase and catalase activity, and decreased ROS levels [41]. In microglial and astrocyte models, trehalose suppressed pro-inflammatory cytokines (e.g., IL-1β, TNF-α) and inhibited inflammasome activation, attenuating neuroinflammatory responses that lead to neuronal injury ain synapse loss in AD [16]. The mechanisms are that trehalose can be used as a dual protection since it can help to clear protein aggregates and protect neurons from secondary damage caused by oxidative and inflammatory stress, both of which are prominent in the AD brain.

### Mitochondrial protection and cellular stress resistance

Dysfunctional mitochondria, impaired energy metabolism, calcium overload and synaptic oxidative damage are the key features of AD pathogenesis that contributes to neuronal degeneration, cognitive decline, and progressive worsening of symptoms. In preclinical models, trehalose has been demonstrated to increase mitochondrial membrane potential, decrease mitochondrial ROS production, and increase mitochondrial biogenesis marker expression [13]. Furthermore, trehalose has been shown to be protective against excitotoxicity and endoplasmic reticulum (ER) stress, due to its stabilizing effect on membranes and protein folding environment, which may be attributed to its chemical-chaperone properties [16]. On a translational level, maintaining the integrity of mitochondria and minimizing cellular stress in neurons affected by AD pathology may provide a means to preserve the integrity of the synapse and postpone functional decline. Table 1 explains some examples of established neuroprotective mechanisms of trehalose in AD, which provides an overview of molecular mechanisms discussed in this section.

**Table 1:** Well-characterized neuroprotective mechanisms of trehalose relevant to alzheimer’s disease

Mechanistic Domain	Key Actions of Trehalose	AD-Relevant Outcomes	References
Autophagy & Proteostasis	Increases LC3-II, reduces p62, enhances autophagosome formation, activates TFEB; may modulate late-stage autophagic flux	Clearance of Aβ, phospho-tau, misfolded proteins; improved proteostasis	[42]
Aβ & Tau Pathology	Reduces Aβ burden; decreases tau phosphorylation; alters APP processing; stabilizes misfolded proteins	Reduced plaque load, improved synaptic markers & cognition	[14]
Oxidative Stress	Activates Nrf2 pathways; increases antioxidant enzymes; reduces ROS	Protection of dopaminergic and cortical neurons from oxidative injury	[41]
Neuroinflammation	Suppresses IL-1β, TNF-α, inflammasome activation; reduces microglial/astrocytic activation	Attenuated neuroinflammation and improved neural survival	[43]
Mitochondrial Protection	Improves membrane potential, reduces mitochondrial ROS, supports biogenesis	Increased neuronal resilience and metabolic stability	[13]

## Underexplored molecular pathways and emerging mechanistic insights

In addition to being an “autophagy inducer”, there is growing evidence for a more intricate and multifaceted pharmacology, involving membrane biophysics, RNA-protein condensates, nutrient sensing, lysosomal signalling, and glial immunometabolism. These pathways are very relevant to AD but are not studied as extensively as other pathways in the research of trehalose.

### Modulation of lipid membrane microdomains and membrane integrity

Membrane lipid microdomains or lipid rafts are central to AD pathogenesis, both Aβ and tau interact with cholesterol and ganglioside-rich rafts, and raft composition is altered in AD cortex, influencing APP processing, Aβ aggregation, and toxicity [44]. This makes any membrane-active property of trehalose mechanistically important. Biophysical work using molecular simulations and spectroscopy has shown that trehalose interacts with phospholipid headgroups and stabilises bilayer structure, reducing dehydration-induced and thermal perturbations [45]. In the context of Aβ, early molecular dynamics studies demonstrated that trehalose weakens Aβ _(29−40)_ peptide insertion into model membranes and reduces peptide-induced structural disruption [46]. More recent localised surface plasmon resonance (LSPR) and biophysical measurements extended these observations to more physiological ternary lipid mixtures (DPPC-POPC-cholesterol), showing that trehalose significantly lowers Aβ binding to membranes and protects bilayers from Aβ-driven damage [17, 18]. Mechanistically, based on current evidence trehalose might exert multiple, partly overlapping effects-headgroup shielding and hydration by forming extensive hydrogen-bond networks with water and lipid headgroups, trehalose increases interfacial hydration, which can reduce the probability of peptide insertion and slow raft coalescence [45, 47]. Suppression of membrane thinning and pore formation**-**simulations indicate that trehalose counteracts Aβ-induced bilayer thinning and pore-like defects, effects that closely correlate with protection against ion leakage and calcium dysregulation key events in synaptotoxicity [17, 46]. Potential modulation of raft organisation though less directly studied, alterations in lipid packing and cholesterol dynamics in the presence of trehalose may partially restore raft architecture disrupted in AD brains, thereby affecting APP localisation and secretase access [17, 44]. From an AD perspective, these data support a possible “membrane-centric” component of trehalose action, although direct experimental validation remains limited in AD. This mechanism is conceptually distinct from autophagic clearance and could be particularly relevant in early, synapse-centric stages of disease when membrane perturbation precedes overt plaque formation.

### Impact on stress granule dynamics and RNA-binding protein regulation

Under stress conditions, the translation of protein gets arrest, which forms membrane-less RNA-protein condensates called stress granules (SGs), as a protective response, that concentrate stalled initiation complexes and RNA-binding proteins (RBPs) including TIA-1, G3BP1 and others [48, 49]. Persistent SG formation and defective phase behaviour have been associated with several neurodegenerative diseases, and AD-related RBPs and tau are known to localise to SGs, hinting at a potential connection between SG biology and tauopathy [50, 51]. A recent study by Dimasi et al. has revealed that trehalose significantly affects SG dynamics without the requirement for autophagosome formation. Trehalose pretreatment resulted in elevated basal levels of phosphorylated eIF2α (p-eIF2α) and its phosphatase subunits, and caused delayed assembly of SGs during acute stress, and accelerated disassembly and recovery of translation following the removal of stress [41, 52]. Such effects were closely linked to regulation of the eIF2α pathway, which is a major point for the integration of various cellular stresses into translational control [48, 53]. In contrast, it is unclear, that whether these mechanisms can be directly activated in the human brain after oral administration of trehalose. This is primarily due to poor systemic and CNS exposure and poor dose determination for BBB penetration. These observations raise several possibilities relevant to AD. On the other hand, persistent SGs can serve as nucleation platforms for aberrant protein aggregation. By shortening SG lifetime and accelerating return to normal translation, trehalose might reduce opportunities for pathogenic co-aggregation of tau, TDP-43, or other RBPs in neurons exposed to chronic stressors [49, 50]. Fine-tuning of eIF2α signalling sustained p-eIF2α and translational repression are observed in AD brain and models, contributing to synaptic failure. Trehalose’s ability to precondition the eIF2α system, yet facilitate dephosphorylation and translational recovery after stress, suggests a hormetic effect that may help neurons cope with repeated insults [52, 54]. RBP trafficking and mRNA fate is through the reshaping SG dynamics, trehalose likely alters the subcellular localisation of RBPs, and the composition of mRNAs retained vs. returned to translation. Although this has not been mapped transcriptome-wide in neurons, SG-associated mRNAs frequently encode synaptic and stress-response proteins, raising the possibility that trehalose indirectly modulates synaptic proteomes in AD-vulnerable regions [51, 55].

### Crosstalk with nutrient-sensing pathways and GLUT/SLC2A transporters

Trehalose is structurally a disaccharide, yet in mammalian systems it behaves in several respects more like a nutrient-sensing modulator than a simple energy source. In a study, trehalose was found to inhibit multiple SLC2A (GLUT) family glucose transporters, including GLUT2, GLUT7, and GLUT9, thereby limiting cellular glucose uptake. This transporter blockade triggered an energy-deprivation-like state, activated AMPK, and induced autophagy, protecting against hepatic steatosis [56, 57]. Follow-up studies identified GLUT8 (SLC2A8) as a specific trehalose transporter required for trehalose-induced autophagy and signalling in hepatocytes. GLUT8-deficient cells did not show the normal AMPK and autophagic response to trehalose, but when the trehalose transporter from an invertebrate was expressed in the cells, these pathways were restored [58, 59]. Recent mechanistic reviews now suggest that trehalose acts in a way in which both carrier-mediated and glucose transporter inhibition generate a “pseudo-fasting” signal that converge on AMPK-ULK1 and TFEB/TFE3, connecting nutrient availability to autophagy-lysosome biogenesis [60, 61].

### How might this nutrient-sensing crosstalk matter for AD?

Although mild AMPK activation and nutrient stress can be neuroprotective, promoting mitochondrial quality control and autophagy. Trehalose-induced GLUT inhibition may lead to restoration of adaptive stress responses, or to further energy deficits if it is too strong, in neurons already insulin resistant and with impaired glucose utilisation in AD. Studies of trehalose and GLUT interactions in direct neurons and direct brain histopathology are lacking and remains to be established. Trehalose could also indirectly affect insulin receptor activity and downstream mTOR signalling by its interaction with insulin and mTOR signalling, pathways that are dysregulated in AD, via its effect on GLUT-dependent glucose flux. This may be used in addition to or instead of the classical mTOR inhibition-based autophagy induction methods [39, 62]. GLUT8 and related transporters are cell-type specific and are expressed in several brain cell types such as glia and neurons. Since the mechanisms by which the brain takes up trehalose are similar to those in the liver, its action on autophagy and metabolism might differ in various brain cell types, which could account for some of the conflicting results observed in different models [58].

### Regulation of lysosome-autophagosome fusion and lysosomal competence

Beyond simply “inducing autophagy,” trehalose has complex effects on lysosomal structure and function. Rusmini and colleagues showed that trehalose rapidly enlarged lysosomes, induced transient lysosomal membrane permeability and promoted calcium release from lysosomes, leading to activation of the calcium-dependent phosphatase calcineurin (PPP3CB). This in turn activated TFEB, a master regulator of autophagy-lysosome biogenesis, leading to increased expression of lysosomal and autophagy genes [51, 63]. More recent work refined this view, suggesting that trehalose exerts a form of “low-grade lysosomal stress” that is sufficient to trigger TFEB-dependent lysosomal biogenesis and restore autophagic capacity in cells with impaired lysosomal function [61, 63]. In parallel, trehalose has been reported to alleviate lysosomal damage and autophagic degradation defects induced by toxins such as swainsonine, further supporting a role in maintaining lysosomal activity [64, 65]. Although activation of TFEB dependent pathways has been demonstrated in in-vitro and in-vivo models, the extent to which comparable effects can be achieved in the human brain remains unresolved. Future pharmacokinetic and exposure-response-based studies are required to estimate whether orally administered trehalose reaches concentrations capable of directly modulating these mechanisms in the CNS [66]. This is highly relevant to AD, where lysosomal dysfunction is now recognised as an early and pervasive pathology in both neurons and microglia. Enlarged, cargo-laden autolysosomes accumulate in dystrophic neurites, and impaired acidification of microglial lysosomes contributes to defective protein degradation and chronic neuroinflammation [61, 67].

Depending on dose and context, trehalose-induced lysosomal stress may either enhance long-term clearance capacity through TFEB-driven biogenesis or if it exceeds, temporary impairment in fusion of autophagosome-lysosome and cargo degradation. This dual mechanism likely explains why some studies report enhanced autophagic flux, whereas others describe impaired late-stage autophagy following trehalose treatment [39, 63]. In addition, this entirely rely on the cell-specific lysosomal responses such as in neuronal cells, microglia, and astrocytes and differ markedly in lysosomal load and function. Whether trehalose’s lysosomal effects are beneficial across all cell types, especially in microglia, the evidence of lysosomal insufficiency in promoting inflammatory phenotype are largely unknown [67, 68]. Because lysosomal membrane dynamics are enriched with cholesterol, the integration of trehalose in membrane activity influences the lysosomal integrity and fusion, which indirectly modulates the autophagic flux and inflammasome activation in the context of AD [45, 61]. Collectively, these observations states that trehalose may function as a context-dependent modulator of lysosomal stress and TFEB signalling, which potentiates the restoration of lysosomal homeostasis which are disrupted in chronic AD pathology.

### Epigenetic, transcriptomic and signalling-cascade effects

Direct evidence for epigenetic modification by trehalose in the brain is limited, but several lines of work support broader transcriptional and signalling reprogramming beyond immediate autophagy markers. As discussed in Section Regulation of lysosome-autophagosome fusion and lysosomal competence, trehalose-mediated activation of TFEB and related MiT/TFE transcription factors may influence a broader transcriptional program extending beyond lysosomal biogenesis [61–63]. This implies widespread changes in gene expression patterns that reshape cellular degradative capacity over time. Studies in peripheral tissues and disease models report that trehalose influences expression of genes involved in oxidative stress responses, mitochondrial function, and inflammatory signalling, often in an AMPK and TFEB-dependent manner [69–71]. In models of neurotrauma and retinal degeneration, trehalose treatment altered expression of pro- and anti-inflammatory mediators, antioxidant enzymes, and autophagy-related genes, consistent with a multi-axis transcriptional response [71–73].

Through the pathogenetic perspectives in AD, several mechanisms such as chromatin and histone modification through metabolic signalling pathways might be influenced by trehalose. This is through the modulation of AMPK and mTOR pathways in the point of view of epigenetics. Trehalose may indirectly affect histone acetylation and methylation patterns, as these nutrient-sensing pathways regulate epigenetic enzymes. While direct experimental evidence for trehalose-mediated transcriptional reprogramming in neuronal circuits is currently lacking, there is a possibility of trehalose in modulating the nutrient sensing pathways which contributes to long-term transcriptional expressions in neuronal networks affected in AD [74–76]. MicroRNA and non-coding RNA regulation in autophagy and lysosomal biogenesis are tightly linked to specific microRNAs. Consequently, trehalose-induced TFEB activation may alter these microRNA networks which influence synaptic plasticity and neuro-inflammatory responses. However, direct experimental evidence which supports this mechanism in AD is currently lacking which highlights a clear research gap for future directions [63, 77–79]. The combination of GLUT inhibition, AMPK activation, TFEB-driven lysosomal gene expression, Nrf2-mediated antioxidant responses, and NF-κB/inflammasome modulation impacts the correlation between trehalose and their relationship to cellular signalling networks that exerts beyond canonical pathways of autophagy. Moreover, extensive research on their functional importance and systems-level signalling in AD remain insufficient. Multi-omics approaches that include transcriptomics, phosphoproteomics, and chromatin accessibility related profiling in trehalose-treated AD models could assist in identifying coordinated pathway changes that are currently uncovered by single-marker studies [16, 80–82].

### Interaction with microglial and astrocytic activation pathways

Neuroinflammation driven by microglia and astrocytes is now recognised as a core driver and amplifier of AD pathology, not just a secondary reaction. Disease trajectory is influenced by microglial activation states, lysosomal capacity and phagocytic clearance of Aβ and synapses, astrocytic reactivity and metabolic support [82, 83]. Trehalose is known to have effects on glial cells that are more than just neuroprotective. Trehalose directly affected glial homeostasis by decreasing microglial activation and restoring disrupted astrocytic cytoskeleton as well as increasing the replication capacity of glial cells in striatal cultures obtained from a Huntington’s disease model [84]. Trehalose decreased the activation of astrocytes and the levels of inflammatory markers in the brain in a lysosomal storage disorder model (MPS IIIB), as well as improving neuronal and retinal pathology [43]. Recently, in an experimental AD mouse model, 4% trehalose was found to suppress the microglial activation marker Iba1 in the hippocampus and cortex, plasma leukocyte elastase activity, and overall, indices of neuroinflammation [85]. The potential of trehalose to regulate microglial autophagy is also highlighted in complementary reviews on traumatic brain injury and other CNS diseases, which indicate its ability to reduce chronic inflammation in the brain and maintain synapses [71, 86]. Mechanisms that may be involved include the role of microglial autophagy-associated phagocytosis in efficient clearance of myelin and protein aggregates; potential enhancement or normalisation of autophagic pathways via the use of trehalose may help to promote clearance of Aβ and damaged synapses while reducing release of pro-inflammatory mediators [63, 79, 86].

**Fig. 2:**
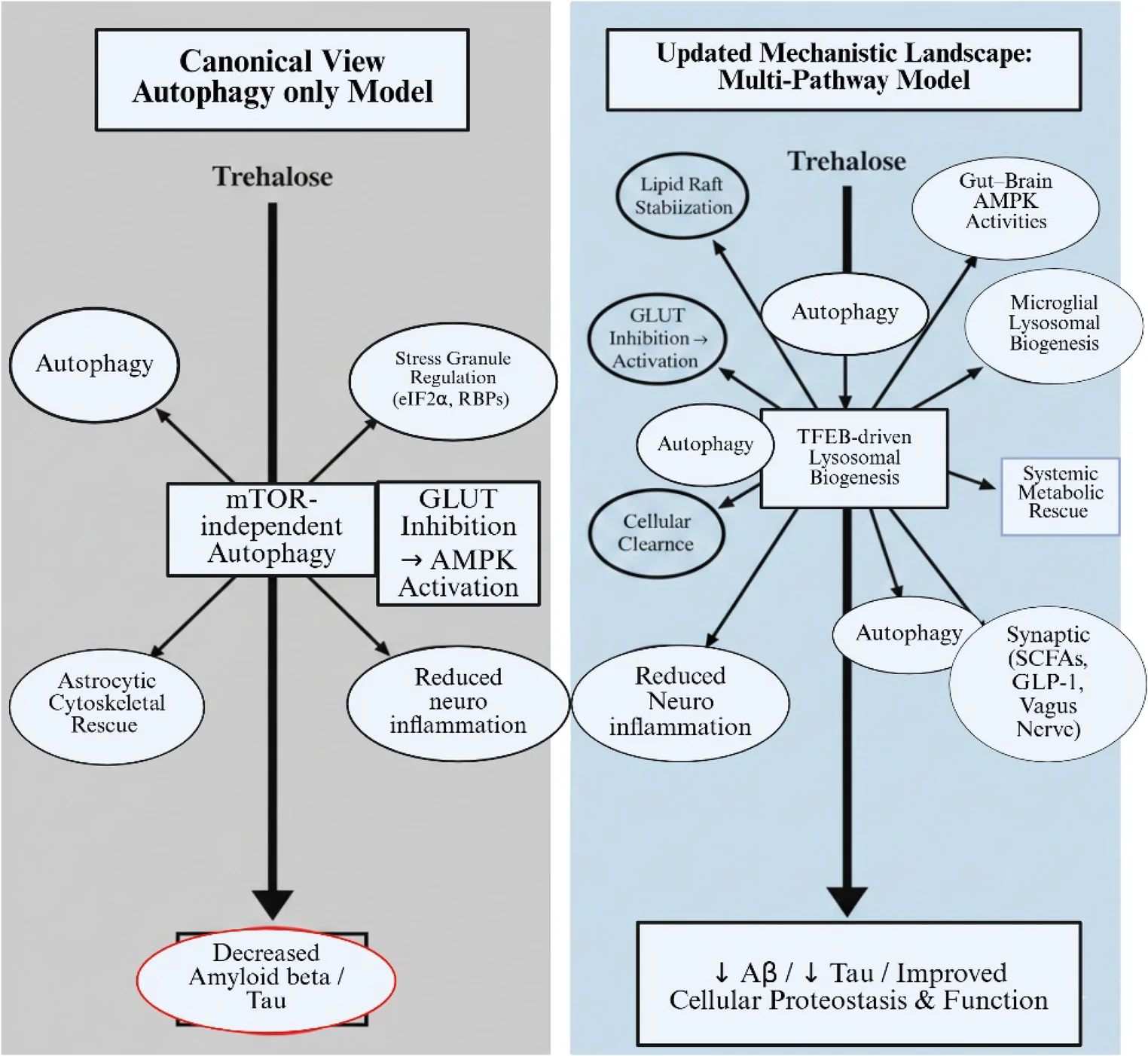
Canonical and emerging mechanisms of trehalose action in alzheimer’s disease

Since the lack of lysosomal function in glia contribute to balancing the inflammatory phenotypes, TFEB activation by trehalose, and restoration of lysosomal function, may help to switch microglia toward a more homeostatic, less neurotoxic state [61, 63, 67]. Regulation of cytokines and inflammasomes by trehalose is through the inhibition of pro-inflammatory cytokines and inflammasome activation in various models, possibly through its antioxidant, lysosomal and metabolic properties. This would directly impact on microglial and astrocytic signalling pathways which regulate the neuroinflammatory tone in AD [43, 87]. While Fig. 2 illustrates the conceptual relationship between canonical autophagy and emerging mechanisms of trehalose action, Table 2 summarises the current evidence supporting these underexplored pathways and their potential implications for AD pathophysiology.

**Table 2:** Emerging mechanistic pathways of trehalose relevant to alzheimer’s disease

Mechanistic Area	Evidence Summary	Potential Impact in AD Pathophysiology	References
Membrane Microdomain Stabilization	Reduces Aβ-membrane binding; stabilizes lipid bilayers; prevents peptide-induced disruption	Protects synapses from membrane toxicity & calcium dysregulation	[61]
Stress Granule Dynamics	Alters eIF2α phosphorylation; delays SG formation; accelerates disassembly	Reduces opportunities for toxic RBP/tau aggregation; restores translation post-stress	[49]
Nutrient-Sensing & GLUT Inhibition	Trehalose inhibits GLUT family transporters, activates AMPK-ULK1 pathways	Induces pseudo-fasting protective signaling; impacts energy metabolism & autophagy	[36]
Lysosomal Competence	Induces TFEB activation via low-grade lysosomal stress; rescues fusion defects	Restores degradative capacity in neurons & glia; improves clearance of aggregates	[80]
Glial Immunometabolism	Reduces microglial activation, enhances phagocytic function, normalizes astrocytic responses	Dampens neuroinflammatory cascades central to AD progression	[86]

## Trehalose and the gut-brain axis

### Overview of gut-brain axis relevance in neurodegeneration

The microbiota-gut-brain axis is a bidirectional communication system between the gastrointestinal system and its associated microbial communities, the immune system, the enteric nervous system, the CNS, and the vagus nerve [88–90]. The composition of the gut microbiota, increased intestinal permeability, systemic inflammation, and imbalances in microbial metabolites are emerging as key factors in the vulnerability of the CNS in context of neurodegenerative diseases, including AD [90, 91]. In human AD cohorts, dysbiosis is linked to the accumulation of Aβ, tau pathology, microglial activation and cognitive decline [90, 92] and in animal models, dysbiosis is related to the accumulation of Aβ, tau pathology and microglial activation [92–94]. In this context, there are promising opportunities to influence neurodegenerative processes via gut microbiota modulation, enhancement of intestinal barrier integrity or changes in microbial-derived signalling.

### Effects of trehalose on gut microbiota composition and metabolites

There are less studies on trehalose than classical prebiotics, but recent research indicates that trehalose can alter the gut microbiota structure and function. Oral trehalose (2% w/v in drinking water) protected against gut microbial diversity reduction, promoted the enrichment of bacterial taxa that are associated with decreased disease severity, and promoted the secretion of glucagon-like peptide-1 (GLP-1) in the gut in a transgenic synucleinopathy mouse model [20]. The results suggest that the neuroprotective properties of trehalose might be at least partially mediated by the microbiota-gut-brain axis, but not through a direct neuronal effect. A recent review notes that trehalose shows prebiotic-like activity, promoting beneficial gut bacteria while protecting intestinal ecosystems under stress [16]. It remains to be determined which specific microbial metabolites (such as short-chain fatty acids (SCFAs), bile acids or microbial-derived neuroactive compounds) are altered by trehalose ingestion in the context of AD models. Given that SCFAs influence microglial activation, neuroinflammation and BBB integrity, the microbial shifts triggered by trehalose may have downstream central effects [88, 91].

### Modulation of gut barrier integrity and systemic inflammation

The integrity of the gut epithelial barrier is key in preventing translocation of microbial-derived endotoxins (for example lipopolysaccharide, LPS) and pro-inflammatory mediators into the systemic circulation, a process implicated in chronic neuroinflammation in AD [93, 94]. Although direct data for trehalose on barrier permeability in AD models is scarce, the microbiota changes it induces suggest a protective effect by enriching taxa associated with mucosal health and reducing pro-inflammatory microbial signals, trehalose may reduce endotoxin load, dampen systemic immune activation and thereby attenuate peripheral-to-central inflammatory signalling. In the synucleinopathy study, trehalose preserved intestinal motility and reduced enteric neuronal loss, outcomes plausibly linked to maintained epithelial-neural-microbiota homeostasis [13, 20, 95]. If confirmed in AD models, this mechanism could underlie part of the neuroprotective effect of trehalose via improved gut barrier function and reduced systemic inflammation.

### Vagus-mediated signalling and peripheral to central immune communication

One of the principal neural conduits of gut-brain communication is the afferent vagus nerve, which senses gut microbial and epithelial signals, relays them to brainstem nuclei, and ultimately influences central neuroimmune, metabolic and synaptic circuits [96, 97]. Trehalose’s modulation of gut microbiota and intestinal hormones (e.g., increased GLP-1) raises the possibility of enhanced vagus-afferent signalling as a mediator of its central effects. In a mouse model, trehalose increased GLP-1 immunoreactivity and prevented reduction of tyrosine hydroxylase immunoreactivity in the enteric and CNS, supporting a gut-initiated neuro-protective axis [25, 98]. From a mechanistic perspective, enhanced GLP-1 or other gut signals may stimulate vagal afferents, leading to activation of central anti-inflammatory pathways, modulation of microglial phenotype and promotion of synaptic resilience. While no studies yet have mapped trehalose-induced vagal activation specifically in AD models, this signalling route is a plausible bridge linking its gut effects to central neuroprotection.

### Linking gut modulation to AD pathology: working model and hypotheses

Gut microbiota dysbiosis and altered gut-brain signalling is increasingly recognised as contributing factors in AD, suggesting that interventions restoring gut homeostasis may affect disease progression. A growing number of human and animal studies demonstrate that AD patients and models show reduced gut microbial diversity, shifts in key taxa, altered metabolite profiles and compromised gut-barrier function [95, 99]. (Table 3) explains the correlation of trehalose and gut-brain axis and its relevance in AD. In germ-free transgenic AD mouse models, the absence of microbiota markedly reduces cerebral amyloid-β (Aβ) load and reintroducing conventional microbiota restores Aβ pathology pointing to a causal role of gut microbes in modulating core AD pathology [100]. Given these data, ingestion of a compound like trehalose may influence AD risk or progression via gut-mediated routes even if its direct brain penetrance is modest. By modulating the microbiota composition and promoting metabolic profiles favouring gut health, trehalose could restore a more balanced microbial ecosystem, reduce pro-inflammatory signalling and support beneficial metabolite production (e.g., short-chain fatty acids, SCFAs), which in turn support gut barrier integrity, dampen systemic inflammation and reduce peripheral immune activation. Emerging reviews highlight that microbial metabolites and dysbiosis-induced systemic inflammation can impair BBB integrity and activate the innate immune cells of CNS, thereby contributing to neuroinflammation, synaptic dysfunction and neurodegeneration in AD [16, 25, 101, 102].

**Table 3:** Proposed gut–brain axis mechanisms of trehalose relevant to alzheimer’s disease

Gut-Brain Pathway	Effects of Trehalose	Relevance to AD	References
Gut Microbiota Composition	Increases beneficial taxa; preserves microbial diversity; promotes SCFA-producing bacteria	SCFAs regulate microglial activation, BBB integrity, and neuroinflammation	[26]
Microbial Metabolites	Enhances GLP-1 secretion; increases butyrate production from trehalose-derived GOS	Supports enteric-vagal signalling and central anti-inflammatory pathways	[80]
Gut Barrier Integrity	Reduces dysbiosis-driven endotoxin leakage; may support epithelial health	Lower systemic inflammation reduces CNS microglial priming	[94]
Vagal Signalling	Trehalose-linked enhancement of GLP-1 may boost vagal afferent signalling	Influences brainstem circuits, microglial tone, and synaptic plasticity	[91]
Systemic Immune Modulation	Reduces peripheral inflammatory mediators; rebalances gut-immune axis	Key bridge between peripheral inflammation and central proteostasis failure	[95]

**Fig. 3:**
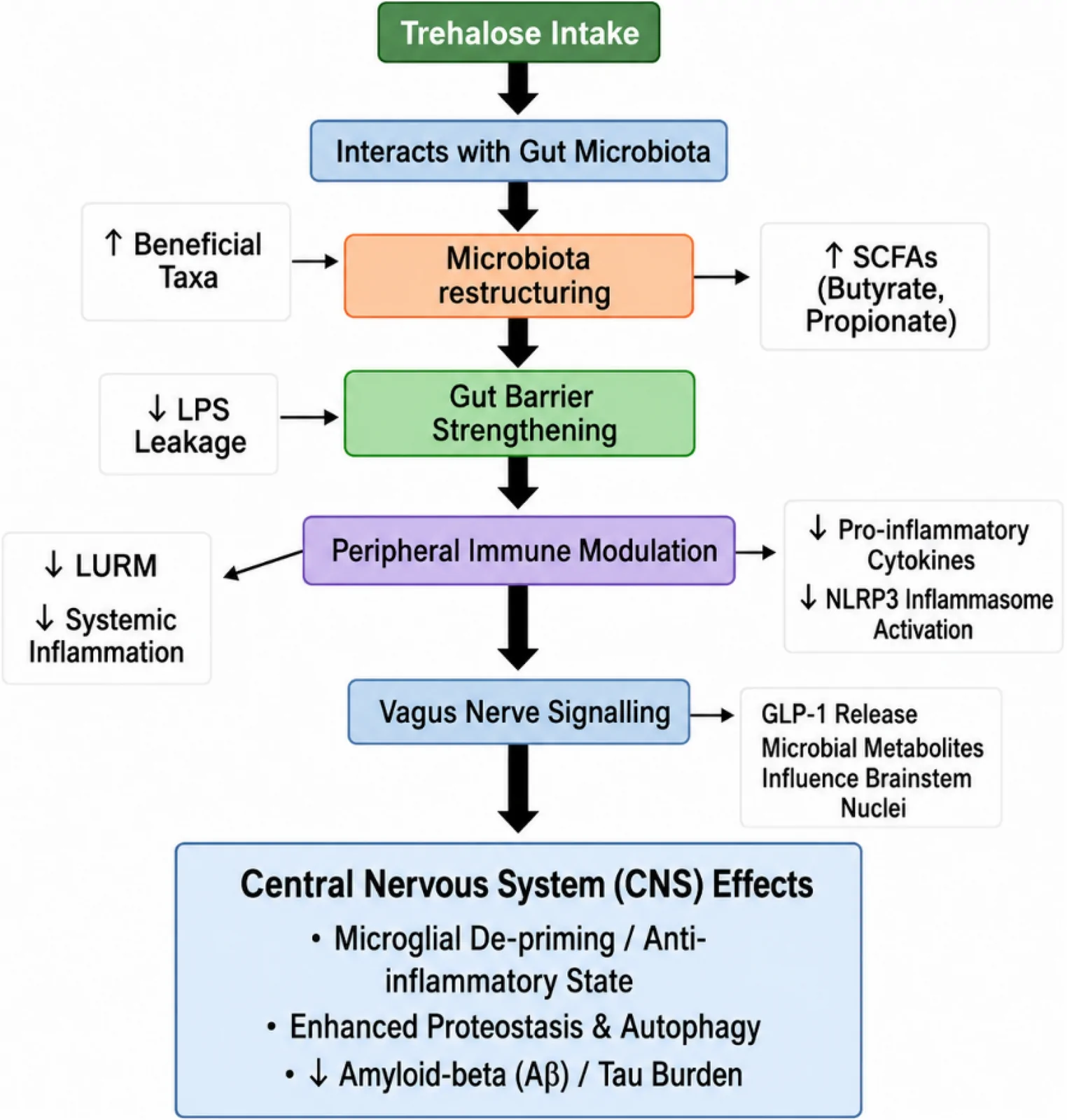
Proposed multistep model of trehalose-mediated gut–brain communication in alzheimer’s disease

Currently, there is no direct evidence that states the importance of gut-brain axis, AD pathology, and trehalose administration. Other models such as germ-free models, faecal microbiota transplantation, microbiota depletion, or vagotomy have not yet been performed in conjunction with trehalose treatment. This might be a research gap for future studies. In this working model, trehalose is hypothesized to influence gut microbiota, which leads to improved gut metabolites, gut barrier integrity, decreased translocation of endotoxin and pro-inflammatory mediators, decreased peripheral immune activation, vagal and humoral signalling to brain, microglial de-priming, decreased neuroinflammation, improved clearance of misfolded proteins and support of synaptic maintenance, and slowing of Aβ/tau accumulation and preservation of cognitive functions [25, 67]. This hypothesis suggests that the neuroprotective properties of trehalose could be more related to its peripheral/gut actions rather than direct actions on neurons, where gut-brain signalling plays a key role in the protective effects of trehalose. This is especially the case because of the low exposure of orally administered trehalose concentrations into the brain, indicating that at least a part of its neuroprotective effects may be mediated by metabolic, peripheral or gut microbiota-related biotransformation processes, and not by direct action on brain parenchyma. However, these mechanistic links have not been directly demonstrated in AD models receiving trehalose. To test this model, the composition of the gut microbiome, levels of metabolites, hormones, markers of barrier integrity, systemic inflammation, and immune and proteostasis readouts in the CNS must be measured in parallel following administration of trehalose, ideally using AD models. The entire pathway from gut to brain needs to be mapped to identify which links are active, which are modulatory and which could be impaired in disease states [103]. (Fig. 3) describes the relationship between trehalose and gut-brain axis and their mechanisms. Therefore, the proposed gut-brain axis model should be regarded as a hypothesis-driven, intended to guide future mechanistic investigations, rather than as an established mechanism of trehalose action in Alzheimer’s disease.

This model represents a conceptual synthesis based primarily on available preclinical evidence; several proposed gut–brain links remain insufficiently validated and should not be interpreted as established causal mechanisms in Alzheimer’s disease.

## Evidence from preclinical and translational studies

### In vitro and in vivo models of AD

Cellular and animal models provide several insights on experimental evidence that trehalose can impact AD-relevant biological pathways. Trehalose has been shown to decrease Aβ and tau aggregation and cytotoxicity in vitro, affecting APP trafficking in parallel, and to modulate markers of autophagy in neuronal cultures and neuroblastoma cell lines. After exposure to trehalose, these effects are accompanied by reduced Aβ secretion and altered APP processing, suggesting that trehalose acts on both down (aggregate clearance) and up (upstream amyloid production) stream pathways [104, 105]. In animal studies, there are several transgenic mouse models that have reported that chronic oral or systemic treatment with trehalose lowers amyloid burden and phosphorylated tau levels, thereby maintaining markers of synapse, and enhances memory-related performance. For example, APP/PS1 and Tg2576 models that exhibited reduced plaque burden and enhanced cognitive function with trehalose treatment [106, 107]. Mechanistically, these effects have been linked to autophagy-lysosome pathway modulation, such as TFEB signalling and lysosomal regulation [13]. Importantly, some in vitro reports suggest that trehalose can also inhibit late-stage autophagic flux under other conditions making it a controversial “autophagy-on” simple effect of trehalose [39].

### Behavioural and cognitive outcomes

Functional correlates of molecular improvements are consistently observed across animal models and effect sizes and reproducibility are variable. Chronic trehalose treatment in Tg2576 and APP/PS1 mice in several studies led to enhanced spatial learning (Morris water maze, radial arm maze) and maintenance of synaptic protein levels, along with decreased amyloid pathology [13, 37, 107]. Other studies have claimed strong rescue of memory with continuous administration of trehalose in drinking water (2–4% w/v), but intermittent dosing or lower doses sometimes fail to show strong or consistent rescue of memory [13, 37]. This behaviour is also seen in non-AD models (Parkinsons, Huntington’s, traumatic brain injury), suggesting a general neuroprotective profile that has functional outcomes. However, there are problems with comparing results across studies because of variations in model, disease stage at treatment initiation, route of administration and behavioural testing protocols, all of which complicate comparisons of optimal treatment to achieve cognitive improvement [105].

### Routes of administration and bioavailability challenges

Translationally critical questions concern the amount of intact trehalose that enters the systemic circulation and the brain following an oral dose. Trehalose is degraded in mammals to glucose in the small intestine by brush-border trehalase, so that only glucose is absorbed and systemic exposure to trehalose is low; some animal studies suggest that some trehalose is systemic, but that its BBB penetration is low and variable [16, 25, 67]. These pharmacokinetic facts suggest two possibilities for the observed CNS effects which includes a peripheral/gut-mediated mechanism (microbiota, metabolites, neuroendocrine signalling) or the requirement for high local/systemic concentrations to obtain modest central uptake. This restriction is often overcome in preclinical studies by increasing the oral dose (2–4% in drinking water), by using parenteral administration or by using genetic/chemical models which increase CNS delivery [93, 102]. To overcome bioavailability issues, formulation strategies have been developed such as the polymer conjugate with trehalose, biodegradable nanoparticles of poly(trehalose) and trehalose-decorated carriers for stabilising trehalose, enhancing its uptake in the mucosa and targeting autophagy-impaired cells. These carriers have demonstrated promising in vitro anti-amyloid activity and stability but are still at the preclinical stage [92, 104]. Safety and metabolic implications of chronic high dosing (particularly glucose load after trehalase hydrolysis) of course also need to be carefully assessed in aged and metabolically compromised subjects prior to human translation [25]. Although numerous studies have reported the modulation of central pathways such as TFEB activation, lysosomal biogenesis, stress-response mechanism of trehalose, there’s a limited literature gaps which are related to bioavailability, dose-response relationship with these mechanisms and limited BBB permeability. However, because of the high rate of hydrolysis of trehalose by intestinal trehalase and its low BBB permeability, it is unclear whether the concentrations reported to be effective in cell models are reached in human brain tissue after oral delivery [101].

### Comparative evaluation with other autophagy-modulating agents

Trehalose is mentioned in the context of pharmacologic and natural autophagy modulators like rapamycin, spermidine, metformin and caloric restriction. Rapamycin (mTOR inhibitor) and spermidine (acetyltransferase inhibitor) strongly promote autophagy and protect in some models of AD, but differ from trehalose in mechanism, side effect profile and systemic effects. Trehalose is usually classified as an mTOR-independent autophagy-inducer (acting through GLUT modulation and/or TFEB activation), which might present a different risk/benefit profile compared to mTOR-targeting drugs [22, 91]. Conceptual comparisons are more common, and while rapamycin has a broad spectrum of effects (immunosuppression and metabolic effects), the multi-layered effects of trehalose (membrane stabilisation, stress granule modulation, GLUT interference, gut-microbiome effects) may offer a variety of neuroprotective actions without the canonical mTOR blockade, with uncertain central exposure [27]. Additive or synergistic effects have been reported for the combination of autophagy enhancers and other complementary mechanisms (e.g., metabolic preconditioning with lysosomal biogenesis activators), but rational combination regimens will need to be carefully dosed and timed to avoid any adverse effect on essential cellular functions [82].

### Integrated perspective and translational gaps

Preclinical data suggest that trehalose is a multi-modal neuroprotective agent, which has been shown to have reproducible molecular and behavioural effects across a range of models, but has not yet been translated to humans due to pharmacokinetic uncertainties, dose feasibility and lack of mechanistic clarity. Rigorous pharmacokinetic studies to quantify intact trehalose in plasma, peripheral tissues and brain following realistic oral dosing, controlled studies to differentiate direct CNS effects from gut-mediated effects (e.g., using trehalase inhibitors, germ-free models, antibiotic-treated models, or gut-restricted formulations); and early pilot human studies to assess tolerability, metabolic effects and biomarker responses (gut microbiome, SCFAs, inflammatory markers, CSF proteostasis biomarkers) [67]. If a reproducible biomarker signal is identified and a favourable safety profile is obtained, then targeted delivery systems or trehalose analogues that are more permeable to the BBB may be considered for proof-of-concept trials. Trehalose is a potential, but not fully validated, translation candidate for AD until then AD [29].

## Therapeutic potential and clinical outlook

### Human trial landscape and safety considerations

While most of the evidence for trehalose in neurodegeneration is from preclinical studies, early human studies and safety data for trehalose is promising. Trehalose has been extensively evaluated for safety and is generally regarded as safe, such as a recent review of safety data showed no consistent adverse effects were seen with any oral treatment or dose, even at doses as high as 50 g in humans [24]. In more recent clinical use, intravenous trehalose was given in high doses (for non-AD use) and early reports indicated that it was well tolerated. In addition to safety, there is early translational interest observed in a Phase I/II study is registered that investigates oral trehalose in humans for neuroprotection (ClinicalTrials.gov) [8]. However, there are no large-scale, long-term, randomized trials that have proven the efficacy of trehalose in AD itself. However, a recent trial in another neurodegenerative disease demonstrated tolerability without any benefit, and tolerability does not equal therapeutic effect. While safety and tolerability are important, there are several potential risks that need to be considered. Trehalose is metabolised by the intestinal enzyme Trehalase, so there may be inter-individual variation in trehalase activity that may affect systemic exposure and efficacy. In addition, chronic high dose consumption can create metabolic stresses (such as a higher glucose load) or imbalance in the gut ecosystem that should be addressed in future human studies. Moreover, microbiota changes need to be considered, and the link between trehalose and the C. difficile strains should be investigated further to prevent the opportunistic pathogens from chronic trehalose supplementation. The available human use data indicate that trehalose is generally safe and well tolerated, although the efficacy data in the field of neurodegenerative diseases, particularly AD, is preliminary. Further systematic and controlled clinical trials are required to determine if the encouraging preclinical characteristic of trehalose translates to clinical utility [106].

### Biomarkers for efficacy assessment

In future AD human trials, strong biomarkers will be essential to track mechanistic engagement and therapeutic effect of trehalose. Proposed mechanisms include proteostasis, autophagy/lysosomal dysfunction, and neuroinflammation, and should be reflected in candidate biomarkers, which should also include possible effects on the gut-brain axis. On the central side, conventional AD markers are still relevant, such as measures of cerebrospinal fluid (CSF) or plasma Aβ (particularly soluble oligomeric species), phosphorylated tau, and markers for synaptic or neuronal damage (e.g., neurofilament light chain, synaptic proteins) can be used to determine the effect of trehalose on pathological protein burden. Peripheral (blood) or CSF levels of lysosomal enzymes or autophagy-associated proteins (LC3, p62/SQSTM1) may be used as an indirect measure of autophagy and lysosomal activity, but sensitivity and validity have yet to be determined. Levels of inflammatory cytokines (e.g., IL-1β, TNF-α, IL-6) in plasma or CSF and markers of neuroimmune activation (e.g., microglial activation imaging, if available), would aid in evaluating immunometabolic effects. Other markers of oxidative stress and mitochondrial function could also be useful given the evidence of antioxidant and metabolic effects of trehalose. Biomarkers in the periphery might include gut-derived metabolites (short-chain fatty acids, bile acids), systemic endotoxin or lipopolysaccharide-binding protein (LBP) levels, gut-hormone levels (GLP-1, PYY), and intestinal permeability (e.g., zonulin, I-FABP) measures, if gut-brain mechanisms play a major role. Concurrently, imaging of the gut (e.g., imaging gut permeability using MRI) or imaging of neuroinflammation (e.g., MRI) or proxies for vagus-nerve activity (e.g., heart rate variability or autonomic function tests) may help provide mechanistic readouts [67]. In summary, a panel of composite biomarkers that encompasses central proteinopathy, autophagy/lysosome, inflammation, metabolism, and gut-brain axis markers would be ideal for assessing the efficacy and mechanism of action of trehalose, particularly in early or prodromal AD, where clinical changes may be gradual.

### Formulation strategies and combination therapies

Translating trehalose into a viable therapy for AD faces challenges, notably limited systemic and brain exposure when given orally. For this, new formulation approaches have been developed. For instance, polymer conjugates containing trehalose have been developed to stabilize trehalose, prevent its hydrolysis in the gut and improve intracellular delivery to cells with defective autophagy or protein aggregation burden [67]. These delivery systems may improve target tissue concentrations, decrease dosing levels, and may even minimize off-target metabolic effects. Combination therapy is another option. However, given that trehalose activates autophagy through mTOR-independent mechanisms, it may be possible to achieve additive or synergistic effects when combined with agents which modulate complementary mechanisms (such as mTOR-dependent autophagy modulators, anti-inflammatories, cholesterol/lipid-modulating drugs, gut-microbiome modulators). Indeed, pre-clinical studies indicate that the use of mTOR-dependent autophagy inducers increases aggregate clearance when combined with the use of the other drugs [8, 106]. Furthermore, the putative gut-brain effects of trehalose could be enhanced by co-administration with gut-targeted interventions (prebiotics, probiotics, and dietary changes). Therefore, trehalose analogues with greater stability (e.g., trehalase resistant) or analogues that are more permeable to the BBB (or selectively targeted to glial/neuronal populations) could represent important translational advances. Initial chemical-biology studies of such analogues are promising but must be carefully evaluated for safety and long-term effects [25].

### Patient stratification and personalized medicine opportunities

It is likely that not everyone or every stage of AD will respond to trehalose therapy in the same way. Tailored interventions can provide optimal benefit and risk reduction. Some of the key stratification parameters may be metabolic status, baseline glucose tolerance, insulin sensitivity, gut microbiome composition, presence of comorbidities (e.g., diabetes, gut disorders), disease stage (prodromal vs. symptomatic), and genetic/epigenetic variants related to the autophagy or lysosomal function. For instance, people with early-stage AD with insulin resistance, gut dysbiosis or metabolic syndrome may reap the maximum benefit from the metabolic, gut-stabilizing, and proteostasis properties of trehalose. On the other hand, if there is a high degree of neuron loss and impaired BBB, trehalose may be less effective unless used in conjunction with targeted delivery. Likewise, individuals who express or have low levels of trehalase (rare but reported in some populations) could experience higher systemic exposure and different metabolic effects. Combination therapy, for example, trehalose with specific pathology-targeting agents (lipid dysregulation, neuroinflammation, synaptic dysfunction) guided by biomarker profiles, could also be employed in precision medicine approaches. Longterm safety surveillance would be important particularly in frail or metabolically compromised subjects. Beyond its role as an autophagy stimulator, trehalose has the potential to simultaneously influence proteostasis, metabolism, gut-brain signalling, and neuroimmune homeostasis, making it a promising therapeutic agent for AD. The realization of this potential will require stringent translational research, controlled clinical trials, assessment of biomarkers, optimized formulations or analogues and patient stratification. If these challenges are overcome, trehalose (or derivatives) may prove to be a new approach to disease modification in AD, especially in early or prodromal stages where multifactorial intervention may help slow or prevent progression [67].

## Future directions

### Key experiments and research priorities

First, it is important to define whether therapeutic levels in brain parenchyma are reached after oral, parenteral and intranasal administration of trehalose, using validated assays for intact trehalose versus metabolites, and which routes are most effective for central autophagic engagement. The studies should be performed in both healthy and disease-model animals, followed by microdialysis or CSF sampling in early phase human trials. Second, the use of standardized dynamic autophagic-lysosomal flux experiments in relevant cell types (neurons, astrocytes, microglia) and in-vivo models is needed, which should be performed using tandem reporters (mRFP-GFP-LC3), pulse chase degradative assays with lysosomal inhibitors, and time-resolved proteostasis readouts to distinguish a rise in autophagosome biogenesis from a true increase in degradative flux. Transient lysosomal perturbation has been shown to be caused by trehalose, thereby underscoring the need for these dynamic assays [79]. Third, head-to-head preclinical comparisons are needed between native trehalose and trehalase-resistant analogues, and encapsulated formulations (nanogels, nucleolipids, poly(trehalose) nanoparticles) to identify if preserved systemic exposure or targeted CNS delivery is superior, while avoiding the metabolic side effects. The experiments should also contain readouts for amyloid and tau clearance, for synaptic integrity, for markers of mitophagy and for markers of microglial phenotype [101]. Fourth, mechanistic interventional trials that modulate the gut microbiome (antibiotics, defined consortia, prebiotics) in parallel with trehalose administration will assess causality of gut-brain signalling and should be accompanied by systemic immune phenotyping and metabolomics to identify peripheral mediators (SCFAs, bile acids, LPS) that may connect gut change to CNS proteostasis. Finally, early-phase human trials should include intensive safety monitoring (glycaemic control, microbiological surveillance for opportunistic taxa, including C. difficile) and include pharmacodynamic biomarkers (CSF autophagy markers, PET, blood NfL) to show target engagement and guide dose selection [101].

Of all the above, another trending research area that is poorly understood is the impact of trehalose in tau seeding and trans neuronal (neuron-to-neuron/synapse-to-synapse) propagation. This is due to current advancements in AD pathology that quantifies the levels of tau present and how it spreads through the brain, in other point of view, this might be similar to prion-like propagation through trans-neuronal pathways. Although several studies have reported reductions in tau burden following trehalose treatment, the evidence remains unclear whether trehalose directly influences the initiation and propagation of pathological tau. Future studies should therefore incorporate assays like tau seeding to determine whether trehalose alters tau aggregation and the seeding competence of pathological tau. In vivo tau propagation models and organotypic brain slice cultures would further enable evaluation of whether trehalose modulates tau release, neuronal uptake, and trans-synaptic spread across interconnected neuronal networks. Integrating these experimental approaches with quantitative assessments of autophagic flux and proteostasis would help distinguish whether reductions in tau pathology result from enhanced intracellular clearance, impaired extracellular propagation, or both. Such studies would provide important mechanistic insight into the disease-modifying potential of trehalose in AD [107].

### Multi-omics and systems-level approaches

Advancing trehalose from mechanistic promise to clinical application requires multi-omic platforms that interrogate the gut–periphery–CNS axis simultaneously. Longitudinal study designs should integrate shotgun metagenomics (strain-level resolution), faecal and plasma metabolomics (targeted SCFAs, bile-acid speciation, LPS-related metabolites), plasma proteomics (inflammation and complement signatures), and transcriptomic or single-cell/nuclei RNA-seq of peripheral immune cells and post-mortem or biopsy CNS tissue where possible. Coupling these layers with phospho-proteomics and lysosome-centred proteostasis readouts will permit pathway-level causal inference and identification of biomarkers predictive of response. Recent human and in vitro trehalose microbiome studies demonstrate that community responsiveness is highly context dependent, underscoring the need for dense baseline phenotyping and repeated sampling to capture temporal dynamics. Systems-level causal modelling using mediation analysis, Bayesian networks, and constraint-based metabolic community modelling can be used to infer which microbial metabolites or immune mediators mediate central proteostatic effects; such frameworks also permit in silico prioritization of combination strategies (e.g., trehalose + defined prebiotic/probiotic or AMPK activator) before costly in vivo testing. Integrative multi-omic signatures will additionally enable biomarker-driven patient stratification (see 7.4), permitting enrichment of clinical trials for biological responders and reducing heterogeneity-driven signal attenuation [106].

### Development of trehalose analogues and targeted formulations

Pharmaceutical innovation should pursue two complementary tracks, which include chemical analogues engineered for trehalase resistance with retained or amplified autophagy-inducing activity, and the delivery systems that protect native trehalose from intestinal hydrolysis and/or target the CNS. Trehalase-resistant analogues such as lactotrehalose and 4-O-methyltrehalose have shown preclinical metabolic and autophagy activity while minimizing concerns related to intestinal breakdown and potential selective enrichment of trehalose-utilising pathogens; systematic medicinal chemistry optimization of these scaffolds to improve potency, selectivity for TFEB/AMPK axes, and ADME profiles is a high priority [99]. Concurrently, formulation science offers multiple translationally tractable approaches such as mucoadhesive intranasal powders or sprays to exploit olfactory/trigeminal routes; polymeric or lipid nanoparticles and nucleolipid carriers that enable sustained release and enhanced parenchymal uptake; and poly(trehalose) or trehalose-bearing polymers that act both as carriers and therapeutic moieties, showing promise in early anti-amyloid aggregation and stability assays. Stability, release kinetics, CNS penetration, immunogenicity, and manufacturability should be evaluated for each modality. Importantly, formulation strategies that will limit systemic exposure to glucose (due to avoidance of hydrolysis by trehalase) will limit metabolic risk in insulin-resistant populations. There are multiple nanocarrier and polymer approaches that have shown preclinical promise and additional regulatory and safety characterization, especially for microbiome disruption and tissue accumulation is required.

## Current limitations

Despite the evidence support the therapeutic potential of trehalose in AD hypothetically, several important limitations currently restrict their clinical and experimental translation through mechanistic interpretation. Though trehalose has demonstrated its neuroprotective action in other neurodegenerative experimental models, the pharmacotherapeutics of oral trehalose and its therapeutic dose concentration within the brain to directly engage intracellular targets such as autophagy, lysosomal signalling, or membrane-stabilizing pathways is unclear and very limited. Certain questions arise when trehalose have the tendency to rapidly hydrolyse by intestinal trehalase together with limited BBB permeability, and how it could specifically target neuronal cells and perform mechanistic action. In addition, there is a need for evidence which justifies the importance of direct neuronal mechanisms compared with indirect peripheral based or gut brain axis related effects. Other proposed mechanisms such as epigenetic regulation, miRNA modulation, and transcriptional reprogramming remain speculative. This is due to the hypothesis that it could be related to AD. Future experiments should therefore integrate quantitative pharmacokinetic analyses with mechanistic studies that could distinguish direct CNS actions from peripheral gut-mediated effects. Multi-omics approaches, microbiome-stratified experimental models, dynamic assessments of autophagic flux, and well-designed early-phase clinical trials which incorporate pharmacodynamic biomarkers would be essential for validating the mechanisms proposed in this review and determining the translational potential of trehalose as a disease-modifying therapy for AD.

## Conclusion

Trehalose is a double-edged sword in the perspectives of AD management. In certain cases, it is as simple autophagy enhancer, in other cases it is viewed as a multifaceted modulator of neuronal and systemic homeostasis. Cellular, animal and emerging translational evidence indicates that trehalose can affect a number of pathogenic domains that are central to AD, such as protein aggregation, lysosomal function, membrane integrity, metabolic stress responses, and neuroinflammatory signalling. Recent studies have extended its mechanistic insights by focusing on the under-explored mechanisms of action on lipid microdomains, stress-granule dynamics, nutrient-sensing pathways, and activation states of glial cells. However, new research on gut-brain biology indicates that orally administered trehalose could also exert its effects outside of the CNS via microbiota-dependent and immunometabolism pathways that impact central vulnerability. Although trehalose has demonstrated neuroprotective effects in preclinical studies, translating it into clinical applications would be challenging because the pharmacokinetic profile of orally administered trehalose has uncertainty regarding direct targeted therapy. In fact, it is difficult to interpret whether the reported intracellular mechanisms observed experimentally could be reproduced in patients with oral treatment. A major challenge for translation is pharmacokinetics limitations, dose feasibility, and the relative contribution of direct versus gut-mediated mechanisms. However, the promising safety profile, dietary feasibility, and mechanistic versatility of trehalose make it a promising candidate for multimodal intervention strategies, especially when used in combination with other complementary therapeutics or advanced formulations. Inter-individual variation in trehalase enzyme expression might influence systemic exposure to intact trehalose, potentially contributing to variability in therapeutic efficacy and limiting the predictability of clinical responses. Future advancements will rely on thorough mechanistic dissections in models relevant to AD, as well as on human studies using biomarkers and on personalised approaches that consider metabolic state, microbiome composition, and disease stage. In the context of these challenges, trehalose and next generation trehalose analogues may provide a useful therapeutic tool, providing a systems level approach to stabilising vulnerable neuronal networks and slowing disease progression in AD.

## Data Availability

No datasets were generated or analysed during the current study.
